# Learning Gain and User Experience of AI Avatar–Based and Human-Presented Explainer Videos: Prospective Randomized Crossover Feasibility Study

**DOI:** 10.2196/90037

**Published:** 2026-06-16

**Authors:** Mike Reichert, Thorsten Jungmann, Ute von Jan, Urs-Vito Albrecht

**Affiliations:** 1 Department of Digital Medicine Medical School OWL Bielefeld University Bielefeld, North Rhine-Westphalia Germany; 2 Faculty of Engineering and Mathematics Hochschule Bielefeld Bielefeld, North Rhine-Westphalia Germany; 3 Peter L Reichertz Institute for Medical Informatics of TU Braunschweig and Hannover Medical School Hannover Medical School Hannover, Lower Saxony Germany

**Keywords:** artificial intelligence avatars, AI avatars, explainer videos, learning gain, user experience, higher education, crossover study, feasibility study, digital learning, artificial intelligence, AI

## Abstract

**Background:**

Explainer videos are widely used in higher education. With the increasing availability of artificial intelligence (AI)–generated avatars, it remains unclear whether the presentation format—human presenter vs AI avatar—affects learning outcomes and user experience, especially in technologically complex fields.

**Objective:**

This study aimed to assess the feasibility of a randomized crossover design to investigate learning gain and user experience associated with content-identical explainer videos delivered by either an AI-generated avatar or a human presenter. Exploratory analyses examined the potential differences between the presentation formats.

**Methods:**

An observer-blinded, prospective randomized crossover feasibility study was conducted with 13 undergraduate engineering students. Participants viewed 2 content-identical explainer videos on fuel cell technology presented by either an AI-generated avatar or a human presenter in a randomized sequence. Learning gains were recorded using a 7-item knowledge test administered at baseline and after the first and second video presentations. User experience was assessed after each video by using the AttrakDiff2 questionnaire. Because there was no washout period and the instructional material was identical in both videos, the second learning phase was vulnerable to carryover and test-retest effects. Consequently, analyses of learning outcomes focused on the initial phase, whereas user experience was examined through pooled comparisons across both conditions.

**Results:**

Both presentation formats were associated with substantial short-term learning gains. The difference in the learning gain between the AI avatar and human presenter videos was not statistically significant (median newly correct items 5, IQR 3-5.5 vs 4.5, IQR 2.5-5; *P*=.51; *Z*=0.66; *r*=0.183). In contrast, user experience ratings were consistently higher for the human-presented video across all AttrakDiff2 dimensions, with small to medium effect sizes. The AI avatar presentation was generally perceived as neutral.

**Conclusions:**

This study shows that investigating AI-based explainer videos vs those using a human presenter in classroom settings is feasible and highlights methodological challenges, particularly those related to crossover designs involving content-identical materials. In this small exploratory sample, no significant differences in short-term learning gains were detected between different presentation formats. Nonetheless, participants clearly preferred human presenters in terms of user experience. These results should not be seen as proof of equivalence but rather as a foundation for future research with larger sample sizes, improved study designs, and more sensitive outcome measures.

## Introduction

### Theoretical Background

Explainer videos are vital in digital education, conveying concepts through audiovisual means [1]. Their importance grows with the rise of online learning, complementing text-based materials especially in self-directed learning. Platforms such as YouTube offer vast, diverse content, but quality varies, and algorithms often prioritize engagement over educational value [2]. Empirical work comparing artificial intelligence (AI)–generated avatar presenters with human presenters in educational videos has only recently emerged. A small but identifiable body of randomized and quasi-experimental studies has compared both formats and converges on the finding that short-term learning gains are broadly comparable across formats whereas user experience (UX) ratings and video engagement tend to favor human presenters [3-7]. A recent rapid review of AI-generated instructional videos synthesizing 15 primary studies from 2023 to 2025 supports this pattern [8]. However, the entirety of this experimental evidence has so far been generated on either subject matter that is broadly cross-disciplinary (general science, technology, engineering, and mathematics introductions and educational technology) or on nontechnical or generic content. No randomized study to date has examined AI avatar–based explainer videos with electrical engineering undergraduate students using domain-authentic content such as fuel cells, circuitry, or signal processing. This is a notable gap because electrical engineering explainer videos are visually dense and schematic centered, introducing a distinct split-attention situation between the presenter and the static technical visualization. There is even eye tracking evidence from Nugroho et al [9] suggesting that avatar presence may be more favorable than physical teacher presence for cognitive load balance in such settings, but this has not been tested in electrical engineering. Therefore, this feasibility study addresses a specific disciplinary, visualization-related, and UX gap rather than a general absence of evidence on AI-generated educational media.

The effectiveness of explainer videos can be understood through established learning theories. Dual-coding theory suggests that information is better retained when processed through both verbal and visual channels, thereby creating complementary memory traces [10-12]. Similarly, the cognitive theory of multimedia learning posits that learners process auditory, verbal, visual, and pictorial information in separate but capacity-limited channels [11,13]. Meaningful learning occurs when learners actively select, organize, and integrate information across these channels. Therefore, multimedia instruction is most effective when it follows principles such as coherence, signaling, and temporal and spatial contiguity [13,14]. As explainer videos combine narration with visual elements, they are well suited to applying these principles. However, their effectiveness may vary depending on presentation format, including whether a human presenter or an AI-generated avatar delivers content. Cognitive load theory further differentiates the demands of multimedia learning by distinguishing intrinsic, extraneous, and germane cognitive load [15]. In domains such as electrical engineering, intrinsic load is often high because topics such as fuel cells involve complex processes, multiple subsystems, and specialized terminology. Therefore, effective instruction should minimize unnecessary extraneous load and support schema construction [15,16]. Presenter modality may influence this balance: human presenters may facilitate comprehension through natural nonverbal cues, whereas AI avatars may introduce additional perceptual or interpretive demands. Beyond cognitive processing, social and experiential factors also shape the impact of educational videos. Social agency theory suggests that social cues—such as a human voice, gaze, and expressive behavior—can enhance engagement and promote active processing by simulating interpersonal interaction [17]. AI avatars represent a form of mediated social presence. Although they can approximate humanlike behavior, perceived artificiality and the uncanny valley effect may reduce acceptance and trust [18]. UX provides an additional perspective on how learners perceive instructional media. According to the framework by Hassenzahl [19,20], perceived quality includes pragmatic quality (PQ; eg, clarity and usability) and hedonic quality (eg, stimulation and identification). In educational contexts, PQ directly supports comprehension, whereas hedonic quality may influence engagement and motivation [21]. However, excessive stimulation may also increase cognitive load if it is not aligned with instructional goals. Taken together, the effectiveness of explainer videos depends on the interaction of cognitive design, social signaling, and UX. This is particularly relevant in technically demanding domains such as electrical engineering, where instructional clarity and learner engagement must be balanced. While AI-generated avatars offer advantages in scalability and flexibility [6,22,23], it remains unclear whether they can match the cognitive and experiential qualities of human presenters, particularly in technically demanding domains.

### Study Aim

The primary aim of this study was to assess the feasibility of a randomized crossover design for evaluating AI avatar–based vs human-presented explainer videos in a classroom setting. Exploratory analyses examined potential differences in learning gain and UX between presentation formats. The findings are intended to inform the design of future larger-scale studies on AI-based instructional media in higher education.

## Methods

### Study Design

This study investigated whether the mode of content presentation in an educational explainer video—via either an AI-generated avatar or a human presenter—affects learning gain and subjective UX among undergraduate engineering students. This study used an observer-blinded, noninterventional, noninvasive prospective randomized crossover design similar to that used in previous studies comparing conventional, book-based learning with learning via a mobile augmented reality app on smartphones [24,25]. This study was designed as an exploratory feasibility study with a primary focus on descriptive analysis and pattern detection rather than confirmatory hypothesis testing. Three knowledge measurements were conducted: baseline (LZ_0_), after the first video (LZ_1_), and after the second video (LZ_2_) following crossover. The crossover structure was chosen for feasibility reasons because it allowed each participant to experience both presentation formats within a small sample and enabled the collection of both learning and UX data for each format in a classroom setting.

### Participants and Randomization

Participants were enlisted from a defined sample population of undergraduate students in the second semester of the industrial engineering program (electronics module) at Hochschule Bielefeld – University of Applied Sciences and Arts. Recruitment took place during regular course sessions in June 2024, and all eligible students present at this time were invited to participate. Participation was voluntary, and no incentives were offered. Formal nonresponse tracking was not conducted; however, all students who agreed to participate were included in the study. Participation required prior written information and implicit consent through questionnaire completion. Participants were randomly assigned to 1 of 2 groups (A or B) and tested in separate rooms to minimize cross-group influence. A facilitator not otherwise involved with the study supervised each group in accordance with a standardized protocol. The sample size was determined pragmatically based on the availability of students within the course setting and the exploratory nature of this feasibility study. No a priori sample size calculation was performed.

### Study Procedure

**Figure 1:**
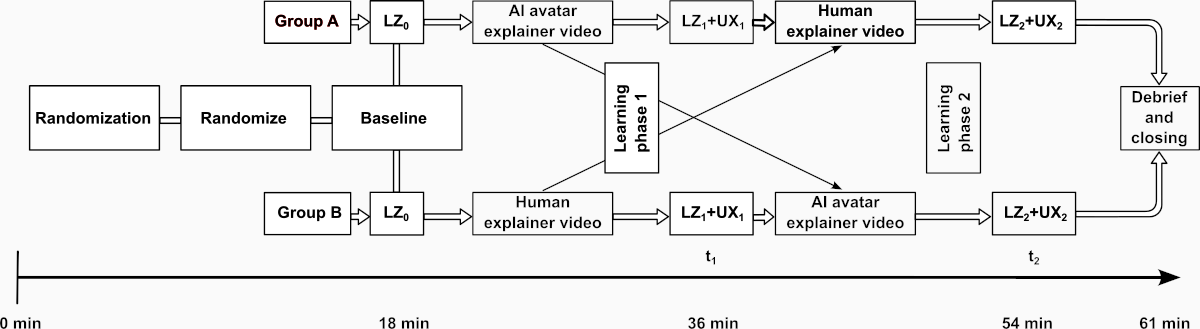
Schematic overview of the randomized crossover study procedure. After providing consent and completing the baseline assessment (LZ_0_), the participants were randomized to group A or group B. In learning phase 1, group A viewed the artificial intelligence (AI) avatar explainer video, and group B viewed the human-presented explainer video, followed by posttest and user experience (UX) assessments (LZ_1_+UX_1_; t_1_). In learning phase 2, presentation formats were crossed over, followed by the second posttest and UX assessment (LZ_2_+UX_2_; t_2_). Approximate durations of each study step are indicated along the bottom of the figure.

At baseline (t_0_), participants generated an anonymous personal code to allow for longitudinal matching of questionnaires. Demographic data (age and gender) were recorded, and baseline knowledge was assessed using a 7-item knowledge test. Group A first watched the explainer video presented by an AI-generated avatar, whereas group B watched first a video with identical content but presented by a human speaker. Immediately afterward (t_1_), knowledge was reassessed (LZ_1_), and UX was measured (UX_1_). Subsequently, presentation formats were crossed over. Group A viewed the human-presented video, and group B viewed the AI avatar video. After the second video (t_2_), learning gain (LZ_2_) and UX (UX_2_) were again assessed (Figure 1).

### Development of the Explainer Videos

**Figure 2:**
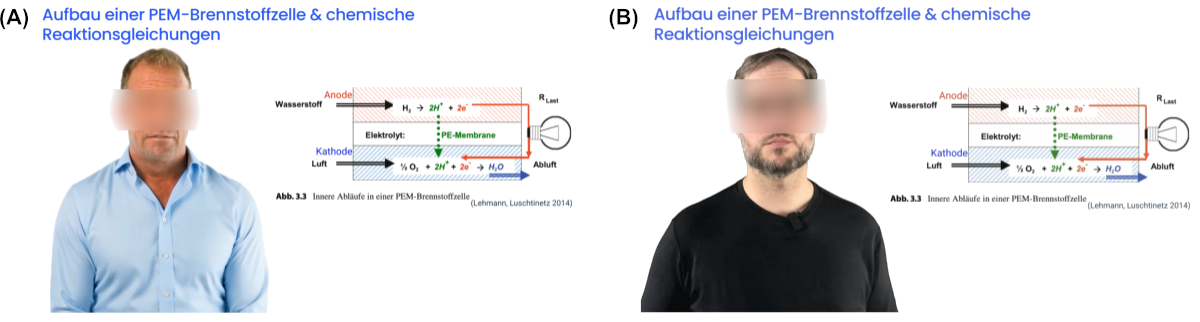
Screenshots from the explainer videos illustrating the structure and operating principle of a proton exchange membrane fuel cell. The facial features are blurred in the screenshots to protect identifying features; the blurring is not part of the videos themselves. The same instructional content and visual background are shown in both versions: (A) explainer video presented by an artificial intelligence–generated avatar and (B) explainer video presented by a human instructor.

A single explainer video (Figure 2) introducing fundamental concepts of fuel cell technology was developed and used in 2 presentation variants. Content structure, learning objectives, visual layout, narration text, scene sequence, duration (5 minutes 25 seconds), and audio quality were kept identical across both versions. The AI-based video was created using the Synthesia platform (Synthesia Ltd), selecting a male avatar with a neutral professional appearance. The video was generated using static background slides and synthetic speech. The human-presented version was recorded in the Hochschule Bielefeld – University of Applied Sciences and Arts learning laboratory using a green screen setup, teleprompter, external microphones (Rode Wireless ME), and a smartphone camera (iPhone 14 Pro; Apple Inc). Postproduction included audio optimization and replacing the green screen with background slides identical to those used in the AI version. Video editing was performed using DaVinci Resolve (version 18.6; Blackmagic Design). The goal was to ensure that the only systematic difference between the videos was the presenter type.

### Learning Gain Assessment

Knowledge was assessed using a self-developed 7-item questionnaire administered at all 3 measurement points (baseline, after video 1, and after video 2). Items were derived directly from the explainer video’s learning objectives and formulated as true-or-false statements with an additional “I don’t know” option. Each correct answer was worth 1 point, resulting in a score between 0 and 7. Learning gain was defined as an increase of at least 1 correctly answered item between 2 consecutive measurement points. A learning regression was defined as a change from a previously correct answer to an incorrect response. The knowledge test was developed specifically for this study and directly aligned with the explainer video’s predefined learning objectives. Each item was designed to assess a key concept presented in the instructional material, ensuring content coverage of the central topics. Given the exploratory, feasibility-focused design, the instrument was intentionally designed as a brief, low-burden assessment to capture immediate learning effects in the classroom. Formal validation procedures, such as expert review, pilot-testing, or psychometric evaluation (eg, internal consistency), were not conducted.

### UX Assessment

UX was measured using the standardized AttrakDiff2 questionnaire based on the UX model by Hassenzahl [26]. The instrument was administered after each video (UX_1_ and UX_2_). AttrakDiff2 consists of 28 semantic differential items rated on a 7-point scale and yields 4 scale scores: PQ, hedonic quality—stimulation (HQ-S), hedonic quality—identification (HQ-I), and overall attractiveness (ATT). The instrument captures both task-oriented and experiential aspects of product perception. Missing values in the AttrakDiff2 questionnaire were not imputed. Scale scores were calculated using the available items for each participant provided that sufficient data were present.

### Analytical Approach

Because of the small sample size and the ordinal nature of the data, analyses were mainly descriptive and exploratory. Learning gain analyses included all 3 assessment points (LZ_0_, LZ_1_, and LZ_2_). Between-group differences in learning gain were assessed using 2-sided Mann-Whitney *U* tests with a significance level of an α value of .05, with effect sizes calculated as *r* = *Z*/√N. Within-group changes between assessment points were evaluated using Wilcoxon signed-rank tests. Because the crossover design used identical instructional material without a washout period, the second learning phase (LZ_1_ to LZ_2_) was considered vulnerable to test-retest and carryover effects. Therefore, the primary comparative analysis focused on the first study period (LZ_0_ to LZ_1_). Analyses involving LZ_2_ were conducted descriptively and are reported in Multimedia Appendix 1 because the second exposure could not be interpreted independently of prior content exposure. For UX outcomes, potential ordering effects were first examined by comparing randomized sequence groups. As no statistically significant ordering effects were detected in this exploratory sample, UX ratings from UX_1_ and UX_2_ were pooled by presentation format for the primary analysis. Phase-specific analyses were additionally conducted as sensitivity analyses and are reported in Multimedia Appendix 1. Given the crossover design and repeated measurements, more advanced methods would ordinarily be appropriate to account for within-subject dependencies and potential carryover effects. However, due to the limited sample size, analyses were restricted to simple nonparametric methods.

All statistical calculations were performed using R (version 4.5.0; R Foundation for Statistical Computing) [27] in combination with the *coin* package (version 1.4-3) [28].

### Ethical Considerations

This study was conducted following the ethical principles outlined in the Declaration of Helsinki. The Ethics Committee of the Ärztekammer Westfalen-Lippe and the University of Münster, Germany, granted ethics approval (reference number: 2024-346-f-N). All participants were informed about the study’s objectives, procedures, and data handling before taking part, and informed consent was obtained before enrollment in the study. Participation was voluntary, and participants could withdraw at any time without consequences. All data were collected anonymously using participant codes created by the participants themselves, ensuring that no personally identifiable information was recorded. Data were handled and stored in accordance with applicable data protection laws to maintain confidentiality and privacy. Participants received neither financial nor material compensation for their involvement in the study.

## Results

### Overview

Feasibility outcomes were assessed across 4 key domains: recruitment, retention, data completeness, and procedural implementation. During regular course sessions, 13 students were approached and screened for eligibility. All were eligible and agreed to participate, resulting in 100% (13/13) enrollment and participation. Once enrolled, all participants completed the study, resulting in a 100% (13/13) retention rate. Study procedures—including randomization, video presentation, and repeated measurements—were implemented as described above. Overall, the study procedures were feasible in terms of implementation, timing, and participant adherence. Of the 13 students recruited for the study, 4 (30.8%) were female (mean age 21.5, SD 3.0 years), and 9 (69.2%) were male (mean age 21.8, SD 3.3 years). The 13 participants were randomized into study groups A (n=7, 53.8%) and B (n=6, 46.2%) and completed the study with no dropouts and complete data for the primary outcome. There were minor missing responses in the AttrakDiff2 questionnaire, as detailed below. In total, 23 items were missing across all participants, primarily affecting the dimensions of HQ-S and HQ-I. However, the number of missing values was limited and did not interfere with scale score calculation. Thus, all 13 participants were included in the final analysis.

### Learning Gain (Primary Outcome; Per-Group Learning Gains)

**Figure 3:**
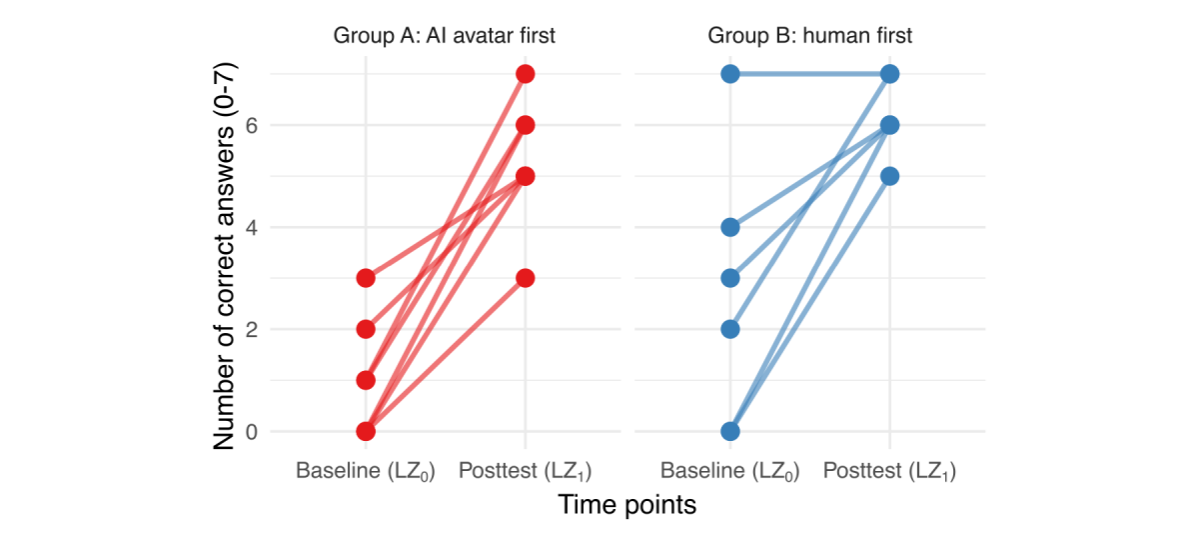
Individual learning trajectories from baseline (LZ_0_) to the first posttest time point (LZ_1_). In group B, participant P1 showed no learning gain because all questions were answered correctly at both measurement points. AI: artificial intelligence.

**Figure 4:**
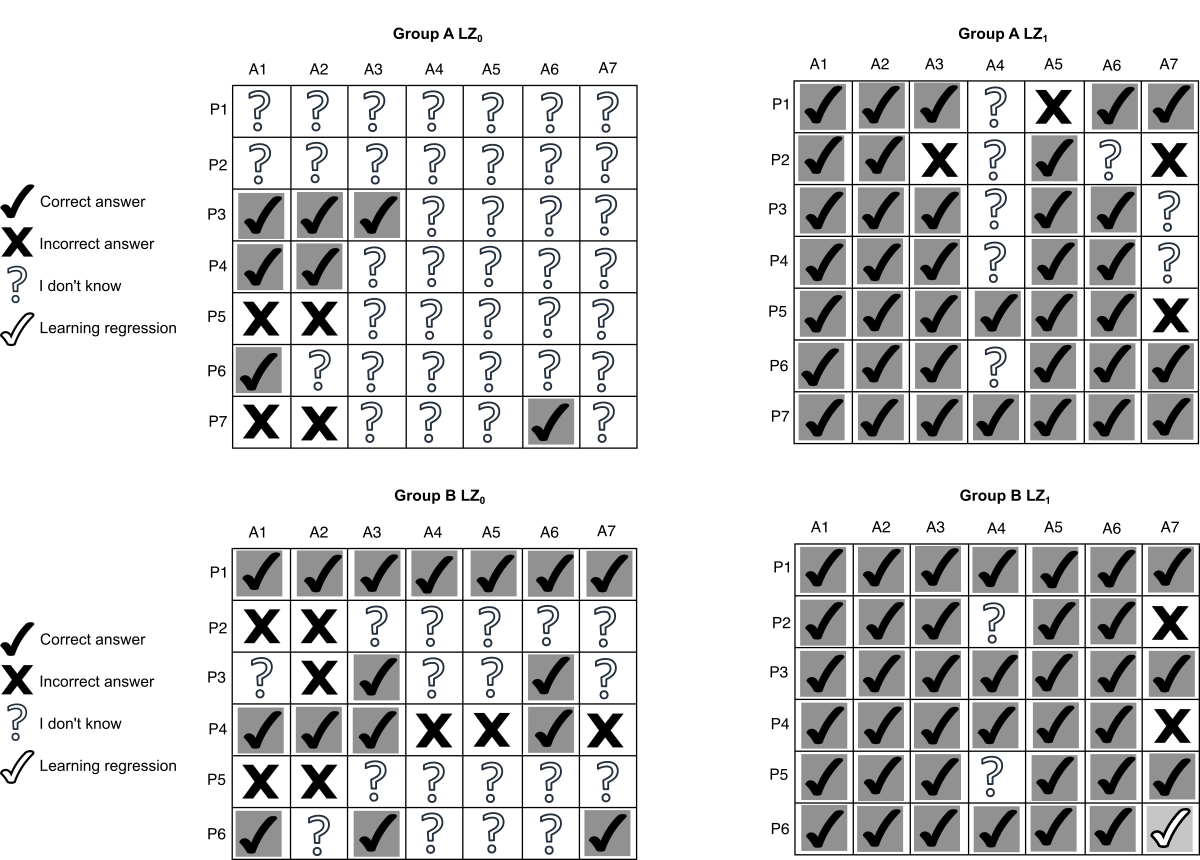
Item-level response patterns for groups A and B across measurement points. Matrices display individual responses (participants P1-P7) to the 7 knowledge items (A1-A7) at baseline (LZ_0_) and after the first explainer video (LZ_1_).

Knowledge scores increased from LZ_0_ to LZ_1_ in both randomized groups, indicating substantial short-term learning within each group after the first video presentation (Table 1 and Figures 3 and 4). The main learning analysis indicates that both the AI avatar and human-presented video were associated with substantial short-term knowledge gains from the pre- to posttest time point, with no statistically detectable between-group difference in the magnitude of gain in this small feasibility sample.

**Table 1:** Relative distribution of the response categories (correct answer, incorrect answer, “I don’t know,” and learning regression from correct to incorrect) at baseline (LZ_0_) and after the first explainer video (LZ_1_)^a^.

Category			LZ_0_, n (%)		LZ_1_, n (%)		Change (%; LZ_0_ to LZ_1_)
**Group A (n=49)**							
	Incorrect	4 (8.2)		4 (8.2)		+0.0	
	I don’t know	38 (77.6)		8 (16.3)		−61.2	
	Correct	7 (14.3)		37 (75.5)		+61.2	
	Regression: correct to incorrect	—^b^		0 (0)		+0.0	
**Group B (n=42)**							
	Incorrect	8 (19.0)		2 (4.8)		−14.3	
	I don’t know	18 (42.9)		2 (4.8)		−38.1	
	Correct	16 (38.1)		37 (88.1)		+50.0	
	Regression: correct to incorrect	—		1 (2.4)		+2.4	

^a^The N values of 49 and 42 correspond to the number of participants (group A: n=7; group B: n=6) multiplied by the number of questions (7 questions per assessment), whereas the n values represent the number of answers for the respective category and assessment (aggregated over all questions).

^b^Not applicable.

At baseline, both groups showed limited prior knowledge, although group B started at a somewhat higher level than group A (Figure 3). From LZ_0_ to LZ_1_, both groups demonstrated clear descriptive learning gains after viewing the first explainer video. This is shown descriptively in Figure 4, which illustrates the progression of responses between baseline and the first posttest time point and highlights learning gains and regressions at the individual and item level. Testing supported the descriptive findings with respect to the learning gains (LZ_1_ – LZ_0_) between the AI avatar group (7/13, 53.8%) and the human presenter group (6/13, 46.2%): there was no significant difference in learning success between the 2 formats (*P*=.51; *Z*=0.66; *r*=0.183). Although UX ratings differed between groups, both presentation formats were associated with comparable short-term learning outcomes. This interpretation was further supported by the number of newly correct items (questions answered incorrectly or marked as “I don’t know” at baseline that were answered correctly at LZ_1_): group A (AI avatar video first) showed a median of 5 (IQR 3-5.5) newly learned items, whereas group B (human presenter video first) showed a median of 4.5 (IQR 2.5-5) newly correct items. Within-group analyses showed that knowledge scores increased from LZ_0_ to LZ_1_ in both randomized groups, indicating significant short-term learning within each group after the first video presentation. For group A (AI avatar video first), median scores improved from 1 (IQR 0-1.5) at baseline to 5 (IQR 5-6) at the posttest time point LZ_1_ (*P*=.02; *Z*=−2.379; *r*=0.636; large effect). Group B (human presenter video first) also showed a substantial increase in median scores from 2.5 (IQR 0-3.75) to 6 (IQR 6-6.75), and again, this was statistically significant (*P*=.03; *Z*=−2.114; *r*=0.610; large effect).

### UX (Secondary Outcome; Evaluation of Sequence Effects)

No statistically significant sequence effects related to the initial presentation mode (group A: AI avatar video first vs group B: human presenter video first) were observed across the AttrakDiff2 dimensions (*P*>.05 in all cases; *r*<0.15 in all cases; Table 2).

**Table 2:** Sequence effects based on a between-group comparison; group A was initially shown the artificial intelligence avatar–based presenter followed by the human presenter, whereas for group B, this order was reversed.

Quality dimension	*Z* score	*P* value	*r*	Magnitude	Group A, median (IQR)	Group B, median (IQR)
ATT^a^	−0.794	.43	0.059	Small	0.0 (–1 to 1.75)	0 (–1 to 1)
HQ-I^b^	1.381	.17	0.104	Small	1.0 (0 to 1.5)	0 (–1 to 1)
HQ-S^c^	−0.394	.69	0.029	Small	0.0 (–1 to 1)	0 (–1 to 1)
PQ^d^	−0.817	.41	0.062	Small	0.5 (–1 to 2)	1 (0 to 2)

^a^ATT: overall attractiveness.

^b^HQ-I: hedonic quality—identification.

^c^HQ-S: hedonic quality—stimulation.

^d^PQ: pragmatic quality.

Following the pooled analysis, the human-presented video was rated significantly more favorably than the AI avatar presentation across all AttrakDiff2 dimensions, with small to medium effect sizes (Table 3). Across all pooled item ratings, the overall comparison between presentation formats remained statistically significant (*P*<.001; *Z*=−6.48; *r*=0.243).

**Table 3:** Comparison of pooled AttrakDiff2 ratings between artificial intelligence avatar and human-presented videos across the 4 quality dimensions.

Quality dimension	*P* value	*Z* score	*r* (effect size)
ATT^a^	<.001	−5.409	0.403 (medium)
HQ-I^b^	<.001	−5.224	0.393 (medium)
HQ-S^c^	.003	2.986	0.223 (small)
PQ^d^	<.001	−5.019	0.379 (medium)

^a^ATT: overall attractiveness.

^b^HQ-I: hedonic quality—identification.

^c^HQ-S: hedonic quality—stimulation.

^d^PQ: pragmatic quality.

### Overview of Presentation Mode–Related Effects (AI vs Human)

**Figure 5:**
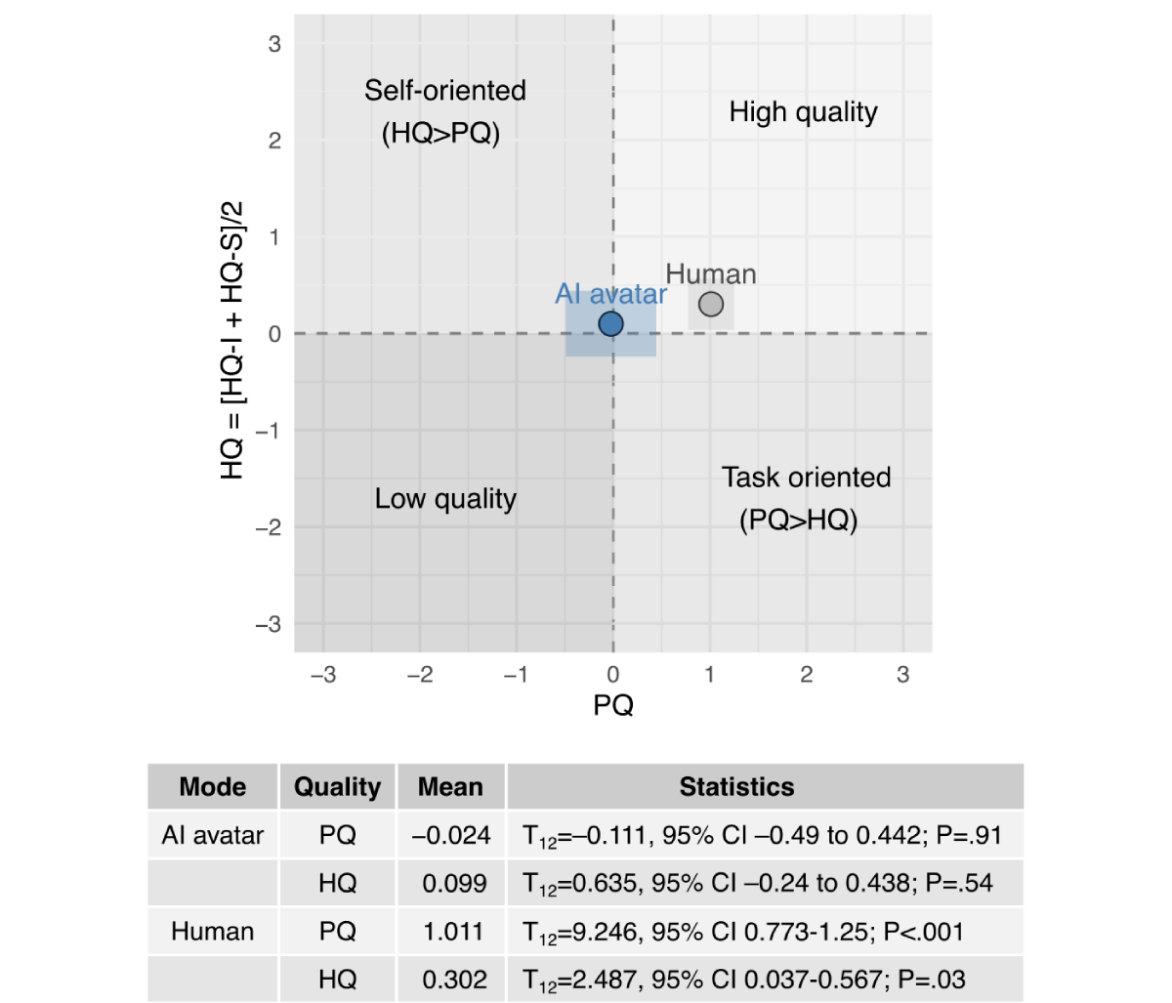
AttrakDiff2 portfolio plot showing pooled presentation format ratings across the user experience assessments (UX_1_ and UX_2_). AI: artificial intelligence; HQ: hedonic quality; HQ-I: hedonic quality—identification; HQ-S: hedonic quality—stimulation; PQ: pragmatic quality.

The AttrakDiff2 portfolio plot (Figure 5) summarizes the character of both presentation modes independent of sequence. The AI avatar presentation mode is categorized as neutral, indicating that, while it was perceived as functional, it failed to deliver a distinctively positive hedonic or emotional experience. In contrast, the human presenter is positioned in the “desired” (high-quality) quadrant. Although this placement is relatively near the border of the neutral zone, it signifies a more balanced and successful integration of usability and user appeal. Additionally, the confidence rectangles for the 2 modes do not overlap. This lack of intersection provides visual confirmation of the statistical divergence between the 2 conditions presented below: the human presenter is perceived as superior in its overall product character compared to the AI avatar.

### Detailed Semantic Differential

**Figure 6:**
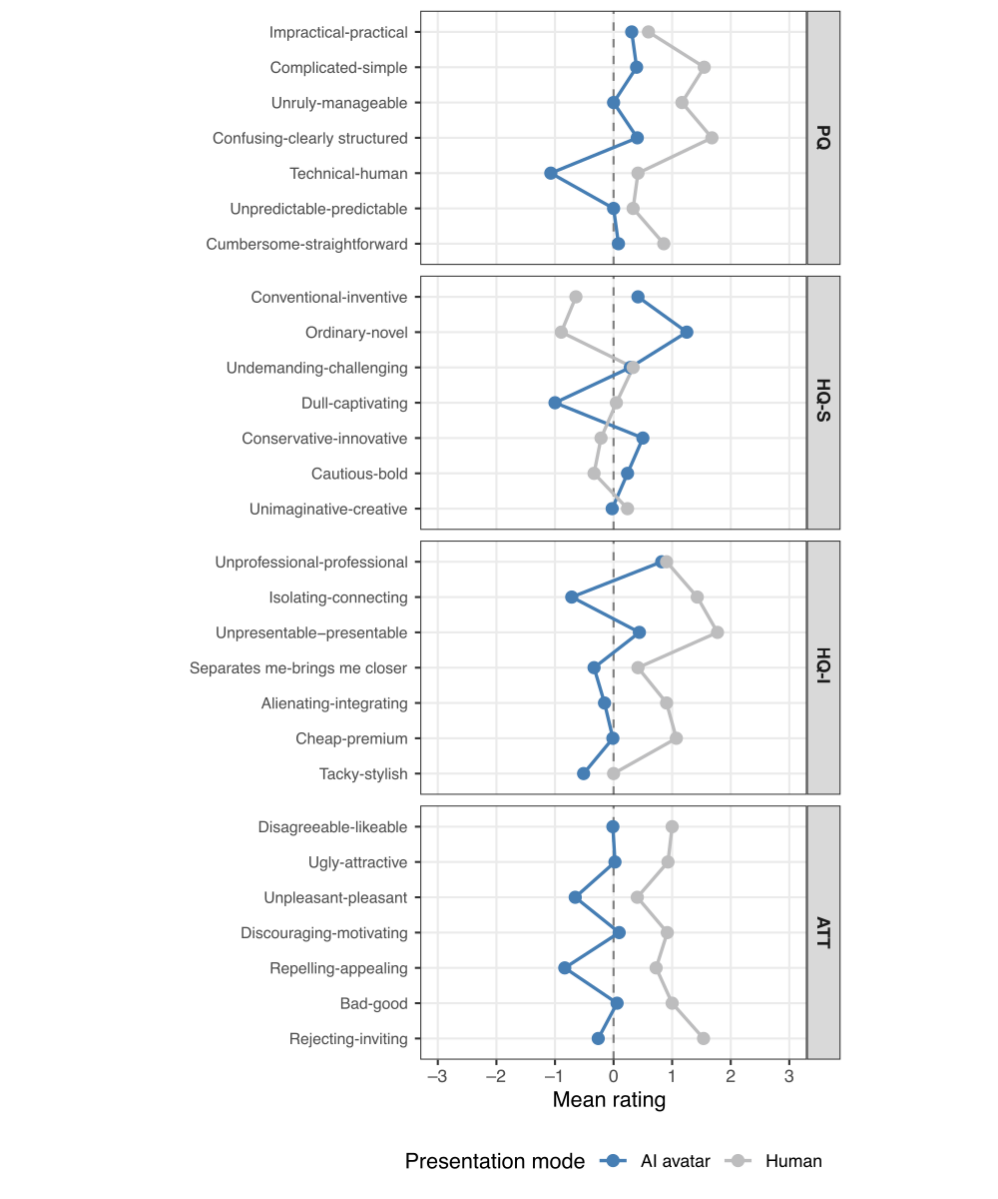
Detailed semantic differential for comparing artificial intelligence (AI) avatar and human presenter–based evaluations from the AttrakDiff2 questionnaire, showing the mean ratings per adjective pair based on the pooled data for user experience assessments UX_1_ and UX_2_. ATT: overall attractiveness; HQ-I: hedonic quality—identification; HQ-S: hedonic quality—stimulation; PQ: pragmatic quality.

The detailed semantic differential (Figure 6) highlights specific adjective pairs where the modes diverged. Notably, for PQ, in contrast to the human presenter, the AI avatar was perceived as rather technical, whereas for HQ-S, it was perceived as novel (with the human being rated as more ordinary) and not quite as engaging as the human presenter (who received neutral ratings on the scale from dull to captivating). However, the results for the other HQ-S items were rather mixed. Across PQ, HQ-I, and ATT, with few exceptions, the human presenter consistently received better ratings than the AI avatar presenter. This possibly explains the smaller effect size for HQ-S compared with the results for the other 3 qualities (with medium effects being observed).

## Discussion

### Feasibility Findings

This study examined how the format of an explainer video—whether presented by an AI-generated avatar or a human presenter—affected short-term learning and UX for undergraduate engineering students. Additionally, it aimed to test the feasibility of using a randomized crossover design in a classroom environment. The results offer a nuanced understanding of both learning outcomes and the overall experience, providing valuable insights into the use of AI-driven instructional media in higher education.

The primary objective of this study was to assess the feasibility of the study design and procedures. Recruitment within a regular classroom setting was successful, with full participation and no dropouts. The randomized crossover procedure, including repeated measurements and format switching within a single session, was logistically implementable and could be carried out as planned. Data completeness was high for the primary outcome, and only a few responses were missing in the UX questionnaire. Overall, the findings suggest that the study procedures were effective for recruitment, implementation, and data collection.

### Learning Gain

The findings are broadly consistent with theories suggesting that well-structured instructional content may reduce the influence of presentation modality on immediate learning outcomes [13,29].

Both presentation formats resulted in notable short-term learning improvements from baseline to the initial posttest time point (LZ_0_ to LZ_1_). Analyses within groups revealed significant gains in knowledge scores for both the AI avatar and human presenter conditions, demonstrating the effectiveness of the instructional materials regardless of the study format. Importantly, there was no significant difference in learning gains between the 2 formats. This outcome supports existing research showing comparable learning results between AI-generated and human instructors [3-7]. A recent rapid review synthesizing 15 primary studies similarly concluded that short-term learning performance tends to be broadly equivalent across presentation formats despite differences in user perception [8]. From a theoretical standpoint, this pattern aligns with the cognitive theory of multimedia learning and cognitive load theory, which highlight that learning outcomes depend primarily on the quality of information processing and the effective management of cognitive load rather than on superficial presentation features [13-16]. As long as instructional content is well structured and aligned with multimedia design principles, different presenter modalities may have only a limited impact on immediate knowledge acquisition [13,29]. Several factors should be taken into account when interpreting these findings. Differences in baseline knowledge between groups may have affected the observed learning gains. Additionally, the knowledge test consisted of only 7 dichotomous items and exhibited ceiling effects after the initial exposure, limiting its sensitivity. Given the small sample size and the exploratory nature of the study, the lack of statistically significant differences should not be seen as evidence of equivalence.

### UX Findings

The results from the UX assessments showed notable, statistically significant differences between the presentation formats, contrasting with the learning outcomes. Participants rated the human-presented video more favorably across all AttrakDiff2 dimensions compared to the AI avatar–based video. The higher scores for PQ indicate that the human presentation was perceived more favorably. This advantage may be due to natural speech patterns, facial expressions, and nonverbal cues, which likely enhance understanding and lessen perceived cognitive load [30]. Furthermore, higher ratings for hedonic quality—specifically, HQ-I and ATT—suggested that the human presenter was perceived as more relatable and socially engaging. These results align with social agency theory, which suggests that humanlike social cues—such as natural voice, eye contact, and expressive gestures—can increase learner engagement and promote a sense of interpersonal interaction [17]. Although AI avatars can mimic certain social cues to some degree, their perceived artificiality may diminish feelings of social presence and identification, especially when subtle mismatches in expression or timing occur [31]. Interestingly, differences in hedonic stimulation were smaller and, in some cases, slightly favored the AI avatar, which could reflect perceived novelty or technological interest. However, this did not translate into higher ATT, indicating that novelty alone cannot compensate for reduced social and pragmatic qualities. This pattern aligns with previous research showing that human presenters are typically rated higher in engagement, perceived quality, and emotional connection even when learning outcomes are similar [4,7]. Although no statistically significant sequence effects were detected in the UX data, caution is warranted in interpreting the results. The small sample size and limited statistical power mean that the absence of clear order effects does not necessarily confirm that they were not present. Subtle carryover or contrast effects might have gone undetected. As a result, combining UX ratings across both measurement points is a practical choice but also introduces some limitations. The lack of significant sequence effects should not be viewed as definitive proof that pooling entirely eliminates order-related biases. While pooling enhances the robustness of comparisons between presentation formats, it may also mask phase-specific effects or interactions between presentation order and user perceptions. Future research with larger sample sizes and designs that explicitly address order and carryover effects is necessary to determine whether pooled analyses truly reflect differences attributable to presentation mode.

### Integration and Implications

A key finding of this study is the observed dissociation between learning outcomes and UX. Although both presentation formats resulted in similar short-term learning gains, participants clearly favored the human presenter from a UX perspective. This difference can be understood through theoretical frameworks that differentiate between cognitive and experiential facets of learning. Cognitive theories focus on efficient information processing as the main factor influencing learning outcomes, whereas UX models emphasize the importance of pragmatic and hedonic qualities in subjective evaluation and engagement [19,32]. The results suggest that a more positive UX does not necessarily lead to greater immediate learning gains. However, UX might still be crucial for longer-term educational factors such as motivation, sustained attention, and ongoing use of learning materials—elements not captured within this short-term investigation. Practically speaking, these findings indicate that AI-generated avatars can effectively deliver instructional content, especially in settings where scalability and resource efficiency are priorities. At the same time, the consistent preference for human presenters underscores the ongoing significance of social and perceptual factors in digital learning environments. This suggests that, while AI avatars may be suitable for standardized or large-scale educational applications, they might be less effective in contexts where engagement, identification, and perceived quality are key to successful learning. Overall, the findings align with existing research showing comparable short-term learning outcomes for both formats alongside a clear advantage in UX for human presenters [3-8]. Our study further extends this research into the domain of electrical engineering—a technically demanding field with high cognitive load and complex visual representations—thereby contributing to an emerging body of evidence in such specialized contexts.

### Limitations

Several limitations should be acknowledged. The most significant one is the small sample size (N=13), which affects statistical power and the generalizability of the findings.

A key methodological limitation of this feasibility study relates to the crossover design without a washout period and the use of identical instructional content across both phases. These conditions introduced substantial risk of test-retest and carryover effects, particularly for the second learning measurement (LZ_2_), thereby limiting its interpretability as an independent estimate of presentation effects. Similarly, UX ratings may have been influenced by comparison or contrast effects between the first and second presentation formats. Although no statistically significant sequence effects were detected in the pooled UX analysis, the small sample size limits confidence in ruling out subtle order-related influences. Consequently, while the crossover design was useful for feasibility purposes, it was suboptimal for isolating presentation format effects on learning and UX.

Finally, this study was conducted in a specific educational setting, which may limit the generalizability of the results to broader contexts.

### Future Work

As this was an initial exploratory feasibility study, the findings offer preliminary insights but cannot support definitive conclusions about comparative effectiveness. Several avenues for future research arise from the results and the identified methodological limitations. First, larger, adequately powered studies are needed to detect small to moderate effects, especially in UX outcomes, where consistent differences were observed. Future research should be based on prestudy power calculations derived from the effect sizes observed in this study. Second, the study design warrants refinement. Due to carryover and test-retest effects noted in the crossover design, future work should consider parallel-group designs, incorporate longer washout periods, and use instructional materials that are similar but not identical to better attribute effects to presentation format. Third, assessment methods for learning outcomes should be enhanced. The brief dichotomous knowledge test used provides limited sensitivity and may suffer from ceiling effects. Future studies should use more nuanced assessment tools such as multiple-choice questions with plausible distractors, transfer tasks, open-ended questions, and delayed posttests to evaluate retention over time. Fourth, research should expand beyond immediate learning outcomes to include motivational and affective factors such as engagement, perceived credibility, trust in AI-generated presenters, and long-term acceptance. Considering the discrepancy observed between learning outcomes and UX, these elements may significantly influence the educational impact of AI-based instructional formats over time. Finally, future investigations should examine a wider range of contexts, including various disciplines, different levels of prior knowledge, and variations in avatar design—such as realism, voice, and gender—to identify the conditions under which AI-generated presenters can be most effectively integrated into educational practice.

### Conclusions

This study shows that implementing a randomized crossover procedure in a classroom setting is feasible, although it also reveals notable methodological limitations when applied to content-identical instructional materials. Both AI avatar–based and human-presented videos resulted in substantial short-term learning improvements, with no statistically significant differences observed between the 2 formats. However, UX consistently favored the human presenter across all measured aspects. These results indicate that while AI avatars can effectively facilitate knowledge acquisition, they currently fall short of human presenters in perceived quality and user satisfaction. As this study was exploratory in nature, further research involving larger sample sizes, refined measurement tools, and optimized experimental designs is necessary to better understand how presentation format influences digital learning outcomes.

## Data Availability

The videos that were used, as well as the datasets generated or analyzed during this study, are available from the corresponding author on reasonable request. However, the complete datasets are not publicly available due to restrictions as they contain information that could compromise the privacy of research participants.

## Appendix

Multimedia Appendix 1 Secondary and sensitivity analyses.

## Abbreviations

AI: artificial intelligence
ATT: overall attractiveness
HQ-I: hedonic quality—identification
HQ-S: hedonic quality—stimulation
PQ: pragmatic quality
UX: user experience
