# Supplementary material for: Learning Gain and User Experience of AI Avatar–Based and Human-Presented Explainer Videos: Prospective Randomized Crossover Feasibility Study
Source: JMIR Form Res. 2026 Jun 16;10:e90037. doi: 10.2196/90037 (PMC13320012; doi:10.2196/90037)

Multimedia Appendix 1. Secondary and Sensitivity Analyses.

The analyses presented below relate to the second learning phase ($LZ_1 \rightarrow LZ_2$) and additional sensitivity analyses from the original crossover design. Because participants were exposed to identical instructional material without a washout period, these findings are considered vulnerable to test–retest and carryover effects and should therefore be interpreted cautiously and descriptively.

Table A.1. Descriptive response patterns for the second learning phase ($LZ_1 \rightarrow LZ_2$). These findings are reported as secondary analyses because the crossover design, without a washout period, introduced a substantial risk of carryover and test–retest effects.

	Category	LZ1	LZ2	Delta
Group A				
	Incorrect	8.2 % (4/49)	4.1 % (2/49)	-4.1 %
	Don't Know	16.3 % (8/49)	8.2 % (4/49)	-8.2 %
	Correct	75.5 % (37/49)	81.6 % (40/49)	+6.1 %
	Regression: Correct → Incorrect	0.0 % (0/49)	6.1 % (3/49)	+6.1 %
Group B				
	Incorrect (0)	4.8 % (2/42)	4.8 % (2/42)	+0.0 %
	Don't Know (1)	4.8 % (2/42)	2.4 % (1/42)	-2.4 %
	Correct (2)	88.1 % (37/42)	92.9 % (39/42)	+4.8 %
	Regression: Correct → Incorrect	2.4 % (1/42)	0.0 % (0/42)	-2.4 %

Figure A.1. Individual response patterns after the second explainer video presentation (LZ_2). Results are shown descriptively because responses may have been influenced by repeated exposure to identical instructional content.

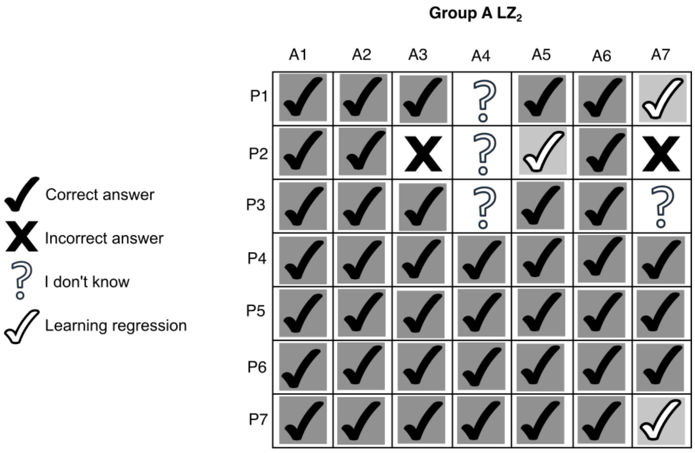

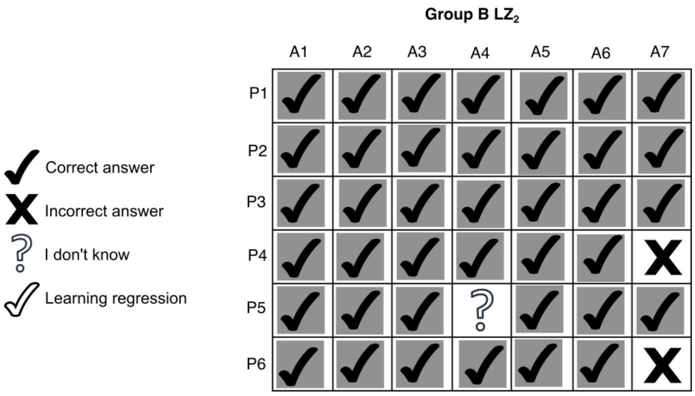

Supplement: Multimedia Appendix 1 [file formative_v10i1e90037_app1.pdf]
